# Potential for reduced premature mortality by current and increased bicycle commuting: a health impact assessment using registry data on home and work addresses in Stockholm, Sweden

**DOI:** 10.1136/bmjsem-2020-000980

**Published:** 2021-01-28

**Authors:** Johan Nilsson Sommar, Peter Schantz, Magnus Strömgren, Bertil Forsberg

**Affiliations:** 1 Department of Public Health and Clinical Medicine, Section of Sustainable Health, Umea University, Umea, Sweden; 2 The Research Unit for Movement, Health and Environment, The Swedish School of Sport and Health Sciences, GIH, Stockholm, Sweden; 3 Department of Geography, Umea University, Umea, Sweden

**Keywords:** cycling, physical activity, death

## Abstract

**Objectives:**

The study aims to make use of individual data to estimate the impact on premature mortality due to both existing commuter bicycling and the potential impact due to increased physical activity through shifting transport mode from car commuting to bicycling.

**Methods:**

Using registry data on home and work addresses for the population of Stockholm County the shortest bicycling route on a network of bicycle paths and roads was retrieved. Travel survey data were used to establish current modes of commuting. The relation between duration of bicycling and distance bicycled within the general population in 2015 was established as a basis for identifying individuals that currently drive a car to work but were estimated to have the physical capacity to bicycle to work within 30 min. Within this mode-shift scenario from car-to-bike the duration of bicycling per week was estimated, both among current and potential bicycle commuters. The health impact assessment (HIA) on mortality due to bicycle commuting physical activity was estimated using the same relative risk as within the WHO Health Economic Assessment Tool.

**Results:**

The current number of bicycle commuters were 53 000, and the scenario estimated an additional 111 000. Their mean bicycle distances were 4.5 and 3.4 km, respectively. On average these respective amounts of physical activity reduced the yearly mortality by 16% and 12%, resulting in 11.3 and 16.2 fewer preterm deaths per year.

**Conclusion:**

The HIA of transferring commuting by car to bicycle estimated large health benefits due to increased physical activity.

**What are the new findings?**

- The study uses travel survey-data and registry-data on home and work addresses to identify 53 000 actual commuting trips currently made by bicycle, with 20 min estimated average bicycle duration.
- Using empirical time–distance relationships among current commuters in the same population an additional 111 000 current car commutes with the estimated physical capacity to bicycle to work within 30 min were identified, with an estimated average bicycle duration of 15 min.
- The study found that among current bicycle commuters the risk of 1-year mortality is on average reduced by 16%, and that the mortality risk would be reduced by 12% among the potential bicycle commuters if they opt to change their mode of commuting from car to bike.

## Introduction

Local measures are taken to increase bicycling in many cities to decrease urban air pollution concentrations and decrease traffic congestion. This also has the additional benefit of increasing the amount of physical activity. Such interventions are of great public health importance since both lack of physical activity and exposure to air pollution are among the leading risk factors for non-communicable disease.^1^ Non-communicable chronic diseases accounts for nearly half of the overall global burden of disease, and 6 out of 10 deaths are attributable to non-communicable disease.^2^

The health benefits of active commuting (transport to work by walking and bicycling) has been summarised in a systematic review of prospective observational studies and intervention studies concluding that active transport has significant effects on morbidity and mortality.^3^ The World Health Organization (WHO) has developed a Health Economic Assessment Tool (HEAT) based on a meta-analysis of mortality in relation to active commuting to estimate the expected reduction in mortality and the economic implication for an increased amount of bicycling and walking in a population. The estimated risk reduction by performing the WHO recommended minimum amount of physical activity was 10% (95% CI 6% to 13%) for bicycling.^4^ This relative risk has been used in several health impact assessments (HIAs) of the benefit of current bicycling and for different transport scenarios.

HIAs of bicycling have been conducted both for scenarios of increased bicycling^5–15^ and to assess health impacts of bicycle sharing systems.^16 17^ The HIAs include health benefits due to increased physical activity, increased air pollution exposure among those that increase their amount of bicycling, reduced air pollution exposure during previous amounts of bicycling and reduced air pollution exposure within the general population. Most of the studies estimated that the majority of the health impact was due to increased physical activity. To our knowledge no previous study has conducted an HIA of increased physical activity based on actual individual commuting trips between home and work. Such individual estimates of physical activity using registry data also enables considering individual differences in bicycling speed.

This study therefore aims to, based on individual registry information on home and work addresses, shortest travel route and empirical bicycling speeds within the study population, estimate the impact on premature mortality due to both existing commuter bicycling and the potential impact due to increased physical activity through shifting transport mode from car commuting to bicycling in case the calculated cycle trip can be undertaken within 30 min or less.

## Methods

### Defining current and alternative modes of commuting

#### Current modes of travel

Based on travel survey data the proportion currently travelling to work with each mode of transport; walking, bicycling, public transport and car, was estimated. To estimate these proportions, individual survey responses were aggregated to small statistical areas within Stockholm County, Sweden. The size of areas was determined by the population density but also taking into consideration natural geographical divisions between areas.

Together with data on traffic flows on roads and estimated proportions of individuals using different modes of travel, the LuTrans transport model for Stockholm County was constructed. Within the LuTrans transport model the travel survey data were used to allocate individual trips to different modes of travel. To obtain the route taken as driver of car, traffic counts was used to allocate car trips to different roads. The model outputs the traffic flow on each road link in the model, where a link is defined as the connection between two major intersections in the road network. LuTrans has been regularly calibrated based on repeated traffic counts.

Using this model all inhabitants (at least 16 years of age) with a home and work addresses within Stockholm County were allocated to a current mode of transport. The construction of the scenario has been described in more detail by Strömgren *et al*.^18^ This allocation also considered individual data on car ownership. Individual information on age, gender, car ownership and home and work address were obtained from the ASTRID database.^19^

#### Alternative scenario

Expected bicycling speeds were based on a previously published study using empirical data on distance and bicycling time within a sample of 455 existing male and female bicycle commuters within the population of Greater Stockholm.^20^ The participants in that study were recruited through advertisements in newspapers. The details of the recruitment and the sample characteristics has been described by Schantz.^21^ The participants drew their own normal bicycle commuting route to work on a map, and its distance was measured using a criterion method.^22^ The bicycling time was measured and self-reported by the participants and was instructed to be without any errands on the way. The procedure has been described in detail by Schantz.^21^ Such a sample of current bicycle commuters may however not represent the time–distance relationship within the general population. Therefore, expected bicycling speeds were scaled down according to the relative difference in maximum oxygen uptake between current bicycle commuters and a sample from the general population. This scaling was performed separately within gender and age groups. Since the general population sample and the sample of current commuters were observed years apart, the relative difference was scaled according to gender specific time trends in body mass index within the population. This scaling of expected bicycling speeds to represent the general population has been described in detail by Schantz *et al*,^20 23 24^ where resulting bicycle speeds estimated by gender stratified linear regressions were given by speed (km/hour)=0.719×(34.8+0.31×age) among men and speed (km/hour)=0.763×(25.9+0.21×age) among women, and where age was measured in years and 0.719 and 0.763 represents the bicycle commuter to the general population effect among men and women, respectively.

This model predicting bicycling speed was used to identify the individuals that have the potential to bicycle to their workplace within 30 min. Registry data on age and gender was retrieved from the ASTRID database. The database also informed about individual home and work address coordinates by which the shortest path along a network of possible roads and bicycle paths was determined. If the individual was estimated to have the potential to bicycle to work within 30 min based on age and gender, and the individual was previously allocated as travelling to work by car, the individual will in the scenario switch to be travelling by bicycle.

### Estimating the amount of physical activity

Using the individual’s shortest path bicycling distance between home and work and the individual’s expected bicycle speed based on age and gender, the bicycling time was estimated. A constant average bicycling speed was assumed. The bicycling intensity was assumed to be 6.8 MET based on measurements for bicycle commuting.^25^ Thereafter the yearly amount (MET-hours/week) of physical activity was estimated assuming four round trips a week 45 weeks a year.

### Health impact calculations

Using the relative risk (RR)-function for all-cause mortality in relation to bicycling used within HEAT,^4^ the reduced number of yearly premature deaths with increased physical activity was calculated. The risk reduction implemented was 10% (95% CI 6% to 13%) for the standardised amount of physical activity corresponding to 11.25 MET-hours/week. This RR is the result of a meta-analysis of seven studies. Six of these studies were based on populations within Western Europe (four from Denmark and one study from UK and Germany, respectively). The bicycling assessed was predominantly commuting. All but one study reported a reduced risk of all-cause mortality with bicycling. The meta-analysis was based on 187 000 individuals observed during in total 2.1 million person-years. The mean age during the follow-up was 56.6 years of age, ranging between 20 and 93 years.

As within HEAT, a straight-line association between physical activity and mortality was assumed with a maximum risk reduction at 447 min of bicycling per week. Age and gender specific mortality data for Stockholm County was used to estimate the impacts on mortality and life-table calculations was used to calculate expected remaining life years based on age and gender.^26^

The public was not involved in the design, or conduct, or reporting, or dissemination plans of the research.

## Results

### Effects on mode of commuting

Of a total of 923 970 current commuters with a home and work address within Stockholm County, approximately 53 000 (6%) were currently estimated to bicycle and 350 000 to be driver of a car (38%) (table 1). The mean age among individuals were 41.8 years and 49% were women. In the scenario that would change to approximately 165 000 bicyclists, an increase to 18% bicycle commuters. This corresponded to approximately 111 000 additional bicyclists. The mean age among the additional cyclists was 42 years and 48% was women. Age-specific number of current and additional bicyclists showed that the proportion of bicyclists less than 35 years of age was higher among current bicycle commuters (table 2).

**Table 1:** Frequencies and proportions of the individuals using different types of transport

Mode of transport	Current situation		Mode-shift scenario		Difference and corresponding proportion	
	Number of individuals	Proportion (%)	Number of individuals	Proportion (%)	Number of individuals	Proportion (%)
Bicycling	53 206	6	164 693	18	111 487	210
Walking	130 441	14	130 441	14	0	0
Public transport	352 412	38	352 412	38	0	0
Car (driver)	352 614	38	241 127	26	−111 487	−32
Car (passenger)	35 297	4	35 297	4	0	0%

**Table 2:** Age-specific number of current and additional bicyclists, average distance and estimated speed and travel time between home and work, and impact on mortality risk and expected number of yearly prevented premature deaths

Age (years)	Number (% of bicyclists)	Distance (km)	Bicycling speed (km/hour)	Travel time (min)	Risk reduction (%)	Number of prevented premature deaths
Current bicycle commuters						
<35	21 662 (41)	4.7	16.2	17.8	14	0.4
36–50	17 736 (33)	4.6	13.5	20.7	17	2.1
>50	13 808 (26)	4.1	10.7	23.3	18	8.8
All	53 206	4.5	13.9	20.2	16	11.3
Additional bicyclists in the mode-shift scenario						
<35	35 945 (32)	4.1	16.5	15.0	12	0.6
36–50	45 485 (41)	3.4	13.6	14.8	12	3.8
>50	30 057 (27)	2.5	10.8	13.8	11	11.8
All	111 487	3.4	13.8	14.6	12	16.2

### Estimated amounts of physical activity from bicycle commuting

**Figure 1:**
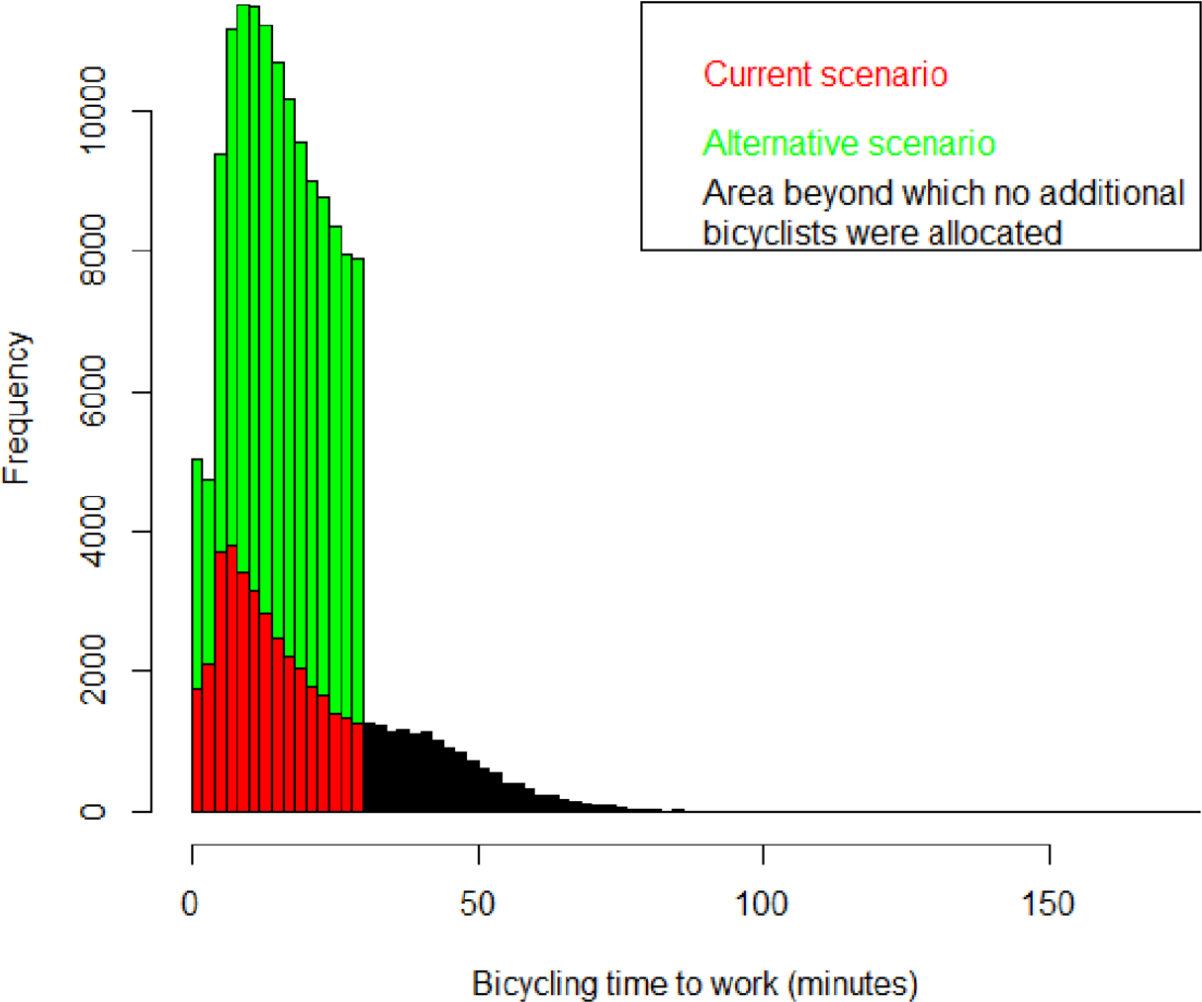
Distributions of expected bicycling times for current cyclists (red and black areas, where the black area indicates the part of the time distribution where no additional cyclists were allocated) and additional cyclists within the car-to-bike scenario (green area).

The mean travel distance to work was 4.5 km among current cyclists compared with 3.4 km among the additional cyclists in the scenario. Estimated current and scenario travel time distributions are presented in figure 1 where the respective mean travel times were 20 min and 15 min.

**Figure 2:**
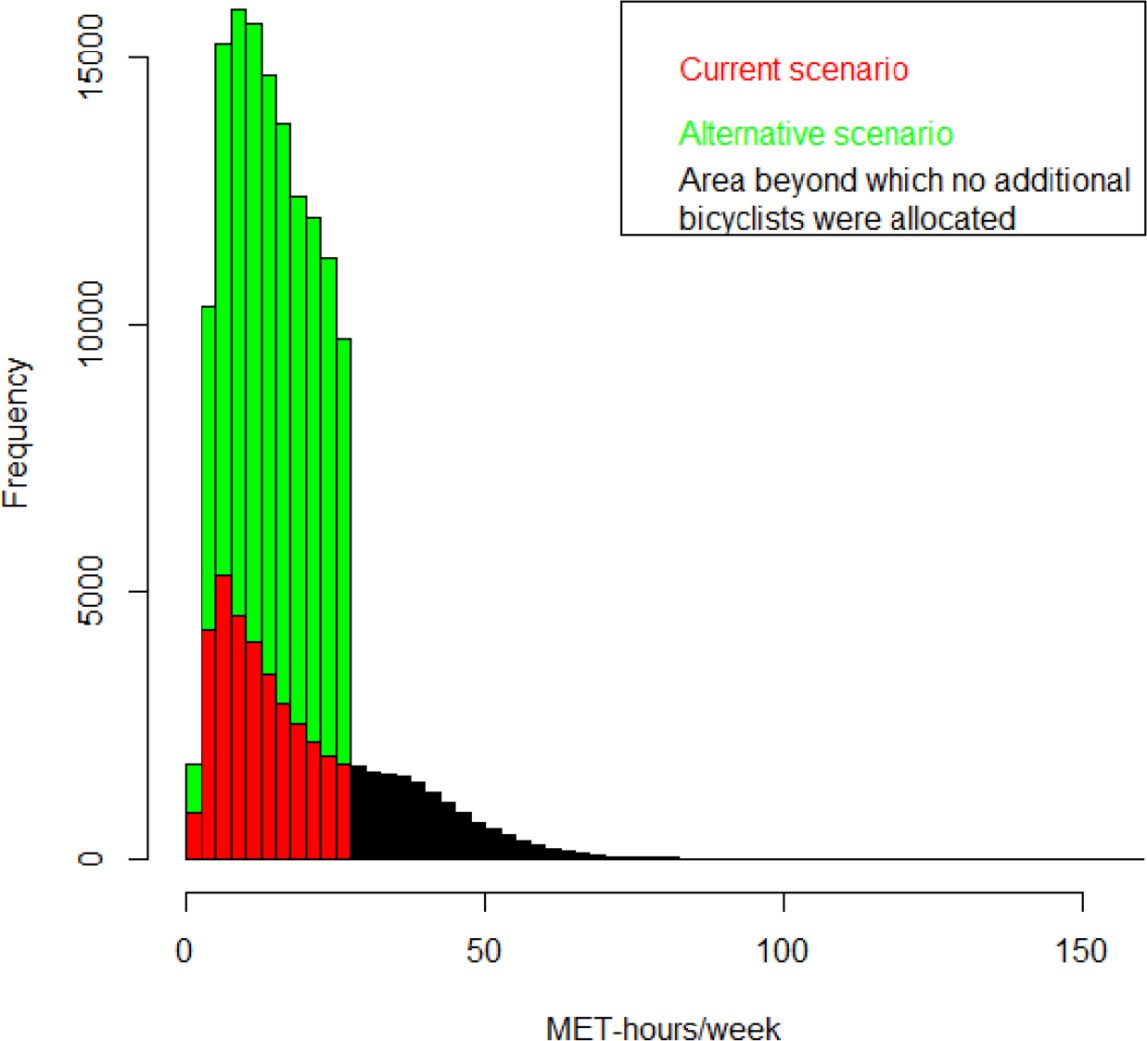
Distributions of expected amount of physical activity achieved among current cyclists (red and black areas, where the black area indicates the part of the distribution where no additional cyclists are present) and additional cyclists within the car-to-bike scenario (green area).

Assuming that the bicycle commuters on average make four round trips per week, 45 weeks a year, with an average bicycling intensity of 6.8 MET, the average amount of physical activity achieved among the additional bicyclists was 13.3 MET-hours/week. The distribution of the increased physical activity was presented in figure 2, where individual amounts of physical activity ranged up to 27.2 MET-hours/week.

### Estimated impact on mortality

**Figure 3:**
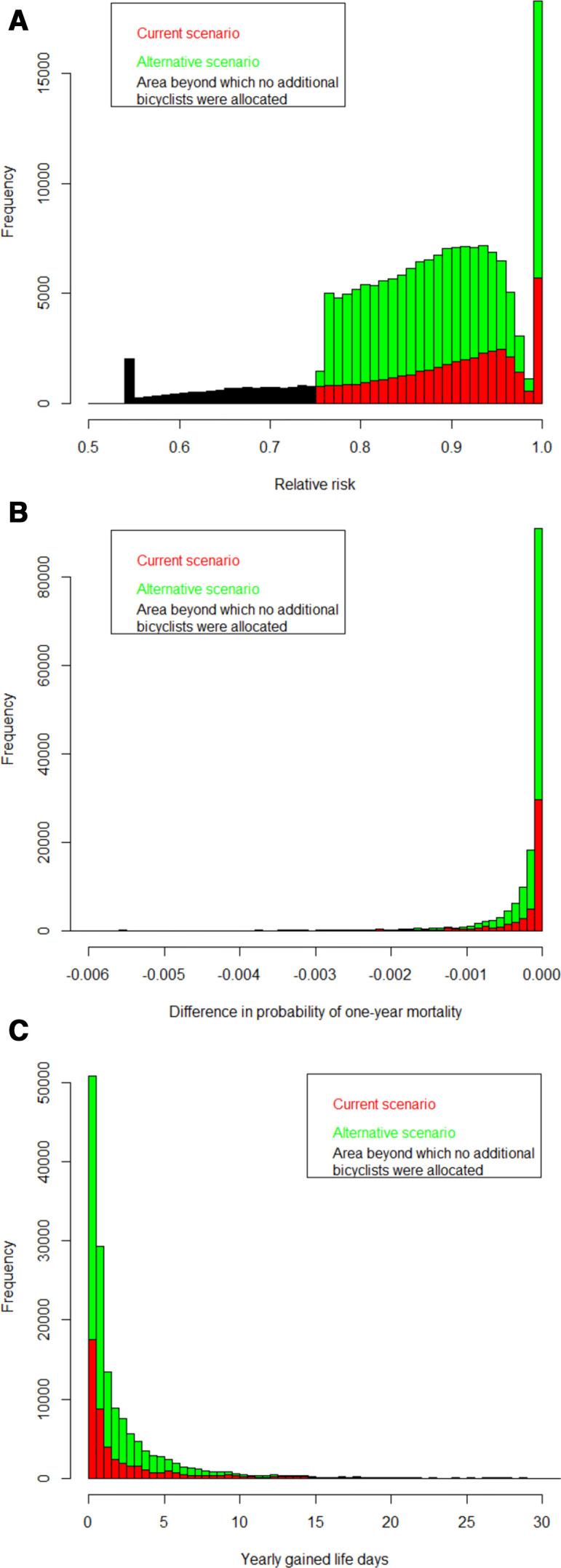
(A) Distributions of relative risks of yearly mortality among current cyclists (red and black areas, where the black area indicates the part of the distribution where no additional cyclists are present) and additional cyclists within the car-to-bike scenario (green area). (B) Distributions of the difference in risk of 1-year mortality comparing driving a car with cycling to work, among current cyclists (red areas) and additional cyclists within the car-to-bike scenario (green area). (C) Distributions of expected yearly gained life days among current cyclists (red areas) and additional cyclists within the car-to-bike scenario (green area).

Based on the RR-function for yearly mortality used within HEAT, with a 10% risk reduction per 11.25 MET-hours/week, the risk of yearly mortality among additional cyclists was reduced by up to 25% (figure 3A) and among current cyclists by up to 45%. The average risk reduction among additional cyclists was 12% and among current 16%. Using registry data on baseline age and gender specific mortality for Stockholm County the resulting impact on mortality for this amount of current and additional bicycling was estimated to be 11.3 and 16.2 yearly prevented premature deaths, respectively (figure 3B). By applying life-table methods these respective number of premature deaths were estimated to correspond to 312 and 469 yearly gained life years (figure 3C). Age-specific risk reductions and expected number of yearly prevented premature deaths showed that 78% and 72% of the expected impact on mortality, for current and additional bicyclists, were among individuals older than 50 years of age (table 2).

## Discussion

This is the first study assessing the health impacts of physical activity in a scenario of increased bicycling based on registry data on home and work addresses. The on average 16% risk reduction for yearly mortality among current bicycle commuters corresponded to 11.3 avoided premature deaths. Additionally, in a scenario where individuals that currently commute by car would start to commute by bicycle if they were estimated to have the individual capacity to bicycle to their workplace within 30 min, the yearly mortality was estimated to be reduced by 16.2 premature deaths each year.

The amount of physical activity obtained from bicycling was estimated based on the bicycling time and intensity, where the bicycling time was estimated based on assumptions about bicycling speed. Woodcock *et al* assumed that the bicycling speed ranged between 12 and 16 km/hour dependent on scenario bicycling infrastructure and waiting times.^15^ Rojas-Rueda *et al* calculated amounts of physical activity based on an average bicycle speed of 14 km/hour.^11^ Previous HIAs have also used similar physical activity intensities. As in the current study, Woodcock *et al*^15^ for instance used 6.8 MET as the average bicycling intensity, and the same bicycling intensity was assumed within each of their scenarios. A similar average intensity was used by Rojas-Rueda *et al*,^11^ 6 MET.

Most commonly previous HIAs have also assumed a linear dose–response between the amount of bicycling physical activity and reduced risk of yearly premature mortality, with a maximum risk reduction of 50% as used by the WHO tool HEAT.^6 15 17 27^ However, the empirical evidence suggest that this association is rather non-linear.^14 28^ Such dose–response functions have been applied in some studies, such as Woodcock *et al*,^15^ but with the added uncertainty about the reference amount of physical activity.

In assessing the health impacts of increased bicycling, it is also necessary to make assumptions of how this affects other physical activity domains. It is possible that increased physical activity through active commuting replaces other types of physical activity, but it is also possible that increased active commuting leads to more physical activity in general. Longitudinal epidemiological studies have found that walking and bicycling add to the total amount of physical activity without reducing other types of physical activity.^29 30^

All previous HIA studies on increased bicycling, or assessments of health impacts of bike sharing systems, have reported great health benefits with reduced mortality due to increased physical activity. According to a review of HIA studies between 12% and 99% of the total impact on health was attributed to increased physical activity.^31^ Lower proportions were reported by Dhondt *et al*,^6^ Grabow *et al*^32^ and Holm *et al*.^7^ The scenario considered by Dhondt *et al*^6^ considered the impact from a 20% increase in fuel price. Expected to increase the number of bicycled kilometres by on average 2%, but with greater increases in public transport, the largest impact was observed due to a reduced risk for injury in traffic accidents and reduced air pollution exposure within the population. Grabow *et al*^32^ estimated health impacts from transferring 50% of car trips <8 km round trip to bicycle. Within the fairly densely populated US region population, and consideration of both fine particles and ozone, almost half of the impact was observed to be due to reduced air pollution exposure within the general population. Increasing the amount of bicycling in Copenhagen, Denmark, Holm *et al*^7^ found that the benefit of increased physical activity would be reduced by two-thirds due to an increase in number of accidents.

The estimated large health impact by reducing premature mortality within this and previous HIAs supports interventions and policies to increase active commuting. The amounts of physical activity through bicycle commuting observed in this study among current, and also estimated among potential additional bicyclists, also indicate that this form of physical activity may reach the 150 min/week physical activity level recommended by WHO. In a review of HIAs of bicycling Mueller *et al*^31^ identified seven studies comparing estimated benefits of increased bicycling to corresponding intervention costs, six of the studies all estimated cost-beneficial effects whereas in one study the result was dependent on the type of intervention considered. The interventions included for instance bicycle infrastructures such as bicycle lanes, encourage use of pedometers and mass media-based community campaigns. As part of the Physical Activity Through Sustainable Transport Approaches project, a review and synthesis of published frameworks of active travel behaviour illustrated examples of pathways to achieve mode-shifts towards bicycling as assessed in the current study.^33^ In their study they for instance highlighted the effects of cycling highway infrastructure where regular bicyclists were affected by gaining more direct, pleasant and safer routes and potential bicyclists by an increased perceived safety that could increase their likelihood to pursue their intention to bicycle or pick up bicycling.

### Strengths and limitations

A strength compared with previous studies is that the study benefitted from the use of individual registry data for the entire study population including home and work address coordinates, which made it possible to perform an HIA of actual commuting trips. Using a network of bicycle paths and roads available for bicycling we were also able to extract the shortest bicycling path between home and work. The individual capacity to bicycle this distance between home and work was assessed by using age and gender specific bicycling speeds based on empirical time–distance relationship data within the study population. A limitation of this assessment is the use of an average intensity (MET-values) for bicycle commuting. This was necessary since studies on bicycle commuting intensities for individual bicycling speeds were lacking. That the values used are reasonable given the average speed applied are supported by recent measurements of bicycle commuting in Greater Stockholm, given that their bicycling velocities were higher.^34^ The usage of an average bicycling intensity affected individual estimates of the amounts of physical activity, however not the average amount or the total impact on mortality. Another limitation was the arbitrary choice of 30 min as the upper limit for the one-way bicycling time between home and work was arbitrary, but the choice aimed to create a reasonably realistic scenario in terms of bicycling time. The average commuter bicycling time in the scenario was found to be considerably lower among the added cyclists compared with current cyclists suggesting that obtained amounts of bicycling could be achievable.

## Conclusion

This HIA using registry data on individual’s home and work addresses, and retrieving shortest travel routes along a road and bicycle path network, estimated large health benefits due to increased physical activity by transferring commuting trips by car to bicycle.

## Data Availability

No data are available.
